# Refining AML Treatment: The Role of Genetics in Response and Resistance Evaluation to New Agents

**DOI:** 10.3390/cancers14071689

**Published:** 2022-03-26

**Authors:** Adriane Halik, Christopher Maximilian Arends, Lars Bullinger, Frederik Damm, Mareike Frick

**Affiliations:** 1 Charité-Universitätsmedizin Berlin, corporate member of Freie Universität Berlin, Humboldt-Universität zu Berlin, and Berlin Institute of Health, Medical Department, Division of Hematology, Oncology, and Cancer Immunology, 13353 Berlin, Germany; adriane.halik@charite.de (A.H.); christopher-maximilian.arends@charite.de (C.M.A.); lars.bullinger@charite.de (L.B.); frederik.damm@charite.de (F.D.); 2 Berlin Institute of Health at Charité-Universitätsmedizin Berlin, BIH Biomedical Innovation Academy, BIH, Charité Clinician Scientist Program, Charitéplatz 1, 10117 Berlin, Germany; 3 German Cancer Consortium (DKTK) and German Cancer Research Center (DKFZ), 69120 Heidelberg, Germany

**Keywords:** AML, mutations, FLT3, IDH1/2, venetoclax, gemtuzumabozogamicin, CPX-351, targeted therapy, precision medicine

## Abstract

**Simple Summary:**

Acute myeloid leukemia (AML) is an aggressive cancer of the hematopoietic system. At present, we know that AML is heterogeneous and varies from one patient to another, often characterized by specific changes in the DNA (mutations). Likewise, we know that the mutational landscape of the disease predicts its response to certain therapies and that it can change under the influence of therapy. Since 2017, the number of potential drugs intended to treat AML has substantially increased and so has our knowledge about the role of certain mutations in the prediction of disease response, relapse and resistance. In this article, we review the current state of knowledge of genetic aberrations with respect to clinical decision making.

**Abstract:**

The number of treatment options for acute myeloid leukemia (AML) has greatly increased since 2017. This development is paralleled by the broad implantation of genetic profiling as an integral part of clinical studies, enabling us to characterize mutation–response, mutation–non-response, or mutation–relapse patterns. The aim of this review is to provide a concise overview of the current state of knowledge with respect to newly approved AML treatment options and the association of response, relapse and resistance with genetic alterations. Specifically, we will highlight current genetic data regarding FLT3 inhibitors, IDH inhibitors, hypomethylating agents (HMA), the BCL-2 inhibitor venetoclax (VEN), the anti-CD33 antibody conjugate gemtuzumab ozogamicin (GO) and the liposomal dual drug CPX-351.

## 1. Introduction

Acute myeloid leukemia (AML) is a genetically heterogeneous and dynamic disease. Currently, AML is classified into three risk groups based on molecular characteristics in the 2017 European Leukemia Net (ELN) risk classification [1]. However, in recent years, rapid progress has been made in describing the molecular diversity and evolution of this devastating malignancy in more and more detail [2,3,4].

Molecular profiling using newly emerging next-generation sequencing techniques has enabled the identification of new genetic lesions and potential therapeutic targets. This growing knowledge has led to the development of inhibitors targeting AML cells with specific mutations, for example, in the *FLT3* [5,6] or *IDH1/IDH2* genes [7,8]. These new treatment options have substantially improved the therapeutic spectrum for patients with respective mutations.

On a separate note, drugs targeting distinct molecular structures such as the BCL-2 inhibitor venetoclax (VEN) [9], the anti-CD33-antibody gemtuzumab ozogamicin (GO) [10,11] and glasdegib, a selective inhibitor of hedgehog signaling [12], have dramatically improved the treatment portfolio for AML patients. As these drugs do not rely on inhibition of a specific oncogenic mutation, but rather pathways, molecular aberrations associated with a response do not necessarily represent the primary drug target but rather a surrogate genetic marker.

Fortunately, comprehensive and often serial molecular profiling of leukemia has become an integral part of clinical studies investigating new treatment options and combination therapies. The generated data sets allow outcome analyses stratified by molecular markers and pave the way towards the goal of truly personalized leukemia therapy. Identification of molecular patterns predicting response and improved survival helps to select patients who would benefit most from a certain drug. Particular attention should likewise be paid to genetic changes associated with primary or secondary treatment failure, as thorough molecular analyses of these subpopulations will help to identify potential future targets for patients with yet unmet therapeutic needs.

**Figure 1:**
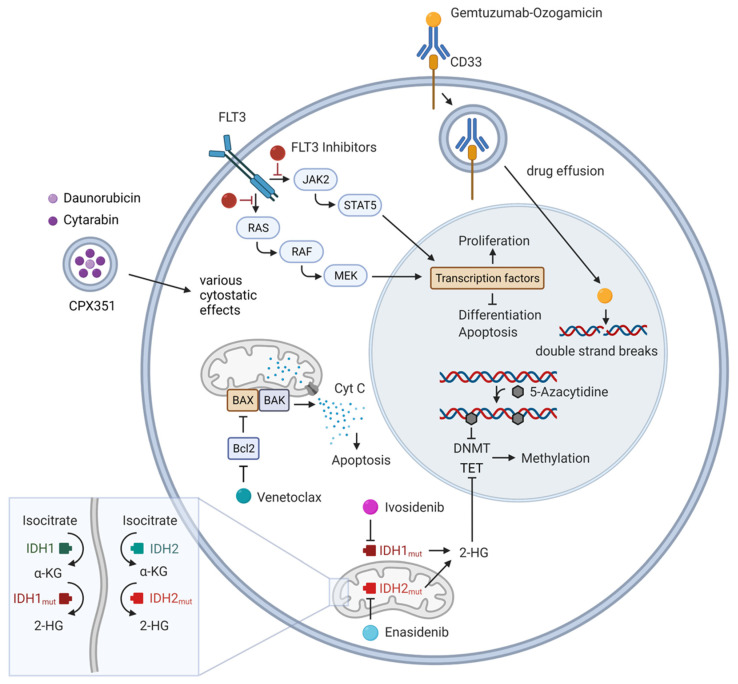
Schematic overview of the therapeutic agents discussed in this article and their mechanisms of action in acute myeloid leukemia. Abbreviations: BAK, Bcl-2 homologous antagonist/killer; BAX, Bcl-2 associated X protein; BCL-2, B-cell Lymphoma 2; DNMT, DNA methyltransferase; FLT3, FMS-like tyrosine kinase 3; 2-HG, 2-hydroxyglutarate; IDH, Isocitrate dehydrogenase; JAK, janus kinase; α-KG, α-ketoglutarate; MEK, mitogen-activated protein kinase; RAF, rapidly growing fibrosarcoma; RAS, rat sarcoma; STAT5, signal transducer and activator of transcription 5; TET, ten-eleven translocation methylcytosine dioxygenase. Created with BioRender.com (accessed on 10 March 2022).

In this review, we therefore report on the genetic background associated with treatment response, primary or secondary resistance, as well as overall survival (OS) for the most frequently used new substances in AML treatment, including FLT3 inhibitors, IDH1/2 inhibitors, the BCL-2 inhibitor VEN, hypomethylating agents (HMA), GO and the new liposomal combination chemotherapy CPX-351 (Figure 1). We focus on substances in broad clinical use that have mainly gained approval by the U.S. Food and Drug Administration (FDA) and/or European Medicines Agency (EMA), acknowledging that there are plenty of promising substances in clinical testing.

## 2. Targetable Gene Mutations

### 2.1. FLT3 Inhibitors

The human FMS-like tyrosine kinase 3 (*FLT3*) gene, located on chromosome 13q12, encodes for a class III receptor tyrosine kinase, which is preferably expressed in myeloid and lymphoid progenitor cells [13]. Once activated by the FLT3 ligand, it promotes cell proliferation and prevents cell apoptosis through PI3K (phosphatidylinositol 3-kinase) and RAS-regulated signaling pathways (Figure 1) [14,15].

**Figure 2:**
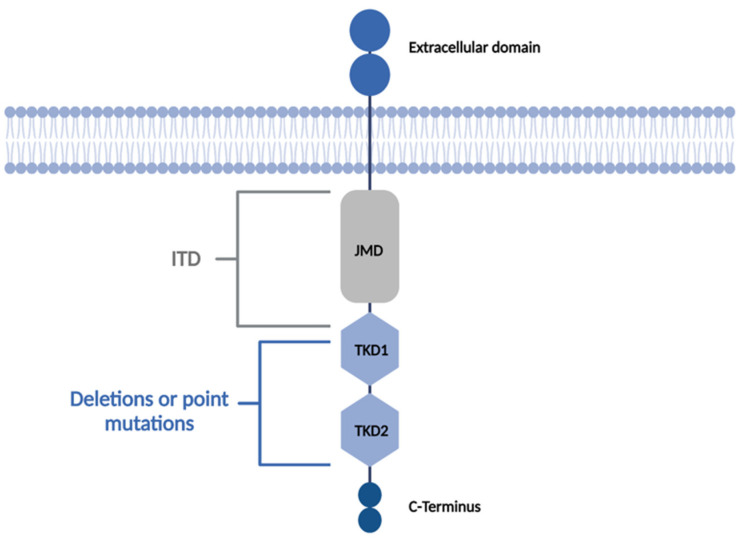
Schematic illustration of FLT3 tyrosine kinase displaying activating *FLT3* mutations according to the affected receptor domain localization. FLT3 kinase comprises an extracellular domain, a transmembrane domain, a juxtamembrane domain (JMD), two tyrosine kinase domains (TKD1 and TKD2) and a C-Terminus. Internal tandem duplications (ITD) are usually located in the JMD whereas deletions and point mutations are mostly found in the TKD domains. Created with BioRender.com (accessed on 10 March 2022).

Approximately one third of newly diagnosed AML patients harbor a *FLT3* mutation and *FLT3* is therefore considered one of the most frequently mutated genes in AML [2]. Activating mutations of the *FLT3* gene most commonly occur as either internal tandem duplications (*FLT3*-ITD) in about 25% of AML cases or as point mutations in the intracellular tyrosine-kinase domain (*FLT3*-TKD) in up to 10% of all AML cases (Figure 2) [16,17]. While *FLT3*-ITD mutations substantially impair clinical outcomes in AML patients, especially when present with a high allelic ratio [18], the prognostic impact of *FLT3*-TKD is still controversially discussed.

Due to the high prevalence of *FLT3* mutations, FLT3 is an attractive target in AML therapy. There are two different classes of FLT3 inhibitors: While type I FLT3 inhibitors (e.g., gilteritinib and midostaurin), bind to the active receptor conformation, type II FLT3 inhibitors (e.g., sorafenib, quizartinib) bind to the inactive conformation of the FLT3 receptor. As consequence, type I inhibitors are effective in *FLT3*-ITD and *FLT3*-TKD mutated AML, while type II inhibitors show little clinical activity in *FLT3*-TKD mutated AML [19,20]. In this review, we will focus on the inhibitors midostaurin, gilteritinib and sorafenib, which are most frequently used in Europe.

#### 2.1.1. Midostaurin

Midostaurin is a multi-target kinase inhibitor, which — among others — targets FLT3 by competitively inhibiting the process of autophosphorylation and subsequent pathway activation [21,22,23]. Midostaurin showed limited single agent efficacy in patients with *FLT3*-mutated relapsed/refractory (r/r) AML not eligible for standard chemotherapy [24]. However, it was approved by the FDA and the EMA in 2017 in combination with intensive chemotherapy (IC) for newly diagnosed AML patients with an activating *FLT3* mutation based on the results of the RATIFY trial [5]. In this phase III trial, the 4-year OS rate was 51.4% in the midostaurin group and 44.3% in the placebo group. Of note, the survival benefit in the midostaurin group was independent across all *FLT3* mutation subtypes.

#### 2.1.2. Gilteritinib

Gilteritinib, a more selective next-generation type I FLT3 inhibitor, is effective in both *FLT3*-ITD and -TKD mutated AML [6,25,26,27]. Clinical efficacy was proven in a multicenter, randomized phase III trial (ADMIRAL trial), that evaluated the benefit of gilteritinib monotherapy against a standard salvage chemotherapy of choice in 371 patients with r/r *FLT3* mutated AML. The trial demonstrated a superior overall response rate (ORR) of 67.6% in the gilteritinib arm versus 25.8% in the standard chemotherapy arm. The most frequent co-occurring mutations in the study population were *NPM1*, *DNMT3A* and *WT1*. Among these, patients in the gilteritinib arm with concomitant *NPM1* and *DNMT3A* mutations benefitted most with a median OS of 10.8 months [6]. Taken together, the higher treatment efficacy combined with a tolerable drug safety profile led to FDA approval of gilteritinib for patients with r/r *FLT3* mutated AML in the United States in 2018 and to EMA approval in 2019.

#### 2.1.3. Sorafenib

Sorafenib is a multi-kinase inhibitor with broad activity against key players of cell proliferation, such as the RAF, KIT and FLT3 kinases. Its antileukemic potency has been evaluated in vitro in diverse *FLT3* mutated leukemic cell lines resulting in reduced cell proliferation, induction of apoptosis and inhibition of FLT3-dependent downstream activities [28]. Recently, sorafenib has been evaluated as maintenance therapy in a phase II trial (SORMAIN) and an open-label, randomized phase III trial in adult patients with *FLT3*-ITD mutated AML undergoing allogeneic hematopoietic stem cell transplantation (HSCT) [29,30]. In the latter, 2-year OS indicated a statistically significant benefit for patients in the sorafenib group with 82.1% versus 68.0% in the control group. Furthermore, sorafenib maintenance therapy significantly reduced relapse risk after HSCT as shown by the 1-year cumulative incidence of relapse being 7.0% in the sorafenib arm and 24.5% in the control arm. In summary, both trials suggest a survival benefit for *FLT3*-ITD mutated AML patients with sorafenib maintenance therapy following HSCT [29,30]. However, sorafenib is not approved for this indication, neither by the FDA nor by the EMA.

#### 2.1.4. Mechanisms of Resistance to FLT3 Inhibitors

While the development of FLT3 inhibitors has markedly improved AML treatment, a substantial proportion of patients show primary treatment failure and resistance or develop a secondary resistance during treatment. Multiple in vivo and in vitro studies give insight into underlying resistance mechanisms against frequently used FLT3 inhibitors. Several mutations associated with clinical outcome and response or resistance to FLT3 inhibitor therapy are depicted in Table 1.

#### 2.1.5. Clonal Evolution

Due to the genetic heterogeneity of AML, the coexistence of *FLT3* mutated and *FLT3* wildtype (wt) leukemia cells is frequent. Most *FLT3* wt leukemic cells show a survival benefit during FLT3 inhibitor treatment and thus may promote relapse depending on their clone size [36].

Based on whole exome sequencing (WES) analyses of serial samples from diagnosis and relapse, clonal evolution patterns of patients with an *FLT3*-ITD mutation treated with midostaurin in the RATIFY trial were identified. Relapse was characterized by either (1) the complete loss of *FLT3*-ITD mutated clones with the emergence of mutations in other pathways, (2) the emergence of a new, midostaurin-resistant *FLT3*-ITD clone, or (3) the persistence of the same *FLT3*-ITD clone from diagnosis, suggesting alternative mechanisms of resistance or insufficient drug levels [32]. These results support the findings of other reports deciphering the evolutional selection under tyrosine kinase inhibitor (TKI) treatment pressure [37].

#### 2.1.6. On-Target Mutations

One mechanism of secondary resistance is the acquisition of new on-target point mutations in the FLT3 kinase domain. First described in blood and bone marrow samples derived from a patient with r/r AML treated with midostaurin, the point mutation N676K was detected within the FLT3 kinase domain and decreased sensitivity to midostaurin was confirmed in vitro [38]. Likewise, other reports indicate the emergence of different types of *FLT3*-TKD point mutations during treatment with sorafenib and quizartinib in patients with *FLT3*-ITD mutant r/r AML (compound mutations) [37,39].

Furthermore, the impact of midostaurin treatment on clinical outcome seems to differ between several *FLT3*-ITD insertion site groups (IS). The addition of midostaurin to IC was associated with an increase in OS compared to placebo in patients with an IS in the juxtamembrane domain with a concomitant *NPM1* mutation (Table 1) [34].

#### 2.1.7. Off-Target Mutations

Other genetic events leading to off-target resistance in FLT3-TKI-therapy have recently been described highlighting the importance of FLT3-independent signaling pathways taking the lead in promoting leukemic cell proliferation (Table 1).

In more than 30% of *FLT3*-mutated r/r AML patients treated with gilteritinib, several new mutations in genes involved in the RAS/MAPK pathway were detected by targeted next-generation sequencing with *NRAS* being the most common mutation finding [33]. Mutations in the RAS-RAF-MEK-ERK pathway seem to preferably occur after treatment with type I FLT3 inhibitors, as revealed by an NGS-based analysis of 67 *FLT3*-mutated AML cases with secondary FLT3 inhibitor resistance performed by Alotaibi et al. [31]. Targeting the RAS-RAF-MEK-ERK pathway therefore seems to be a reasonable way to improve response rates. In the latter study, other frequently acquired off-target mutations were found in genes involved in epigenetic modification (*DNMT3A, TET2, IDH2*) [31], including a targetable lesion in case of an *IDH2* mutation. However, it should be noted that acquisitions of RAS/MAPK pathway mutations have also been reported for other myeloid malignancies treated with various TKIs such as ruxolitinib [40] or imatinib [41] suggesting a recurrent mechanism of clonal selection/ evolution.

The occurrence of *WT1* mutations, which have been associated with inferior outcomes in some but not all studies [42,43,44,45], has been described after treatment with midostaurin or gilteritinib at relapse or disease progression [32,33]. Furthermore, in a few studies, cytogenetic changes such as *BCR-ABL1* fusions have been identified on FLT3-TKI-treatment at relapse or refractory disease [33,46]. The de novo acquisition or expansion of activating *JAK* mutations also seems to play a pivotal role in the development of FLT3-TKI resistance. In 6 out of 136 *FLT*-ITD-positive AML patients who underwent FLT3-TKI treatment, distinct point mutations in *JAK1, JAK2* and *JAK3* were detected in samples obtained after treatment or at disease relapse [35].

### 2.2. IDH Inhibitors

Isocitrate dehydrogenases (IDH) are enzymes catalyzing the oxidative decarboxylation of isocitrate to α-ketoglutarate (α-KG). Its two isoforms *IDH1/IDH2* are recurrently mutated in approximately 20% of de novo AML [2]. Mutations are found at conserved residues (*IDH1* R132; *IDH2* R140, R172) and result in a neomorphic function of the enzyme, catalyzing the reduction from α-KG to the oncometabolite 2-hydroxyglutarate (2-HG) [47], a competitive inhibitor of α-KG-dependent enzymes including the ten-eleven translocation (TET) family of 5-methylcytosine hydroxylases (Figure 1). The accumulation of 2-HG leads to epigenetic dysregulation and impaired hematopoietic differentiation [48,49,50].

*IDH* mutations are early events in the evolution of preleukemia to overt AML. In contrast to other mutations affecting key players of epigenetic transcription regulation such as *DNMT3A, TET2,* or *ASXL1*, *IDH* mutations rarely occur as clonal hematopoiesis of indeterminate potential (CHIP), and thus represent an attractive target for AML therapy [51,52]. Therefore, small molecule inhibitors were developed that allosterically inhibit the respective mutated enzyme isoform, leading to a reduction in 2-HG levels and induction of cell differentiation (Figure 1) [53,54,55].

#### 2.2.1. IDH1

In 2018, the IDH1 inhibitor ivosidenib was approved by the FDA for the treatment of patients with r/r *IDH1*-mutated AML [7] followed by approval by the FDA for patients with newly diagnosed *IDH1*-mutated AML not eligible for IC [56] in 2019. Approval was based on the results of a phase I open-label single-arm multicenter study demonstrating the safety and efficacy of ivosidenib. In the r/r setting, complete remission (CR)/complete remission with incomplete hematologic recovery (CRi) rate was 30.4% and the median OS was 8.8 months [7]. In the group of patients with newly diagnosed *IDH1*-mutated AML not eligible for IC, CR/CRi was achieved in 42.4% with a median OS of 12.6 months.

Predictors of response/resistance to IDH1 inhibition were assessed by Choe et al. in a subset of 174 patients of this study through paired genotyping at the beginning of treatment and at relapse/progression [57]. Baseline co-mutations in the receptor tyrosine kinase (RTK) pathway (in particular *NRAS, PTPN11*) were associated with a lower chance of achieving CR/CRi as a best response, while *JAK2* co-mutations were associated with a higher rate of response (Table 2). No significant correlation between *IDH1* mutation VAF (variant allele frequency) and response was found. Analysis of cases with initial response revealed two main mechanisms of secondary resistance: the emergence of clones with RTK pathway mutations and the emergence of 2-HG restoring mutations, which are either isoform-switching (*IDH2* mutations) [58] or second-site mutations within *IDH1* (e.g., mIDH1-S280F, most probably preventing drug/cofactor binding), leading to increased 2-HG levels (Table 2) [57]. Isoform switching as a mechanism of acquired resistance under IDH inhibition has previously been observed in a case series by Harding et al. [58].

#### 2.2.2. IDH2

For *IDH2*-mutated AML, the IDH2 inhibitor enasidenib was approved by the FDA in 2017 for the treatment of r/r AML on the basis of a phase I/II study [8]. The study included a total of 200 patients with r/r *IDH2*-mutated AML. Under treatment with enasidenib, an ORR of 40.3% was achieved, and the median OS was 9.3 months. Enasidenib also showed moderate efficacy as a monotherapy in a phase I/II study of 39 older patients with newly diagnosed AML [64] resulting in a CR/CRi rate of 21%, an ORR of 30.8%, and a median OS of 11.3 months.

Stein et al. analyzed remission and response patterns in 125 patients treated with enasidenib on a molecular level [60]. No significant differences in response between mutations in codon R140 and R172 were found. Clearance of IDH2 clones was associated with achievement of CR and in patients who achieved CR, baseline *IDH2* VAF was significantly lower in *IDH2* R140 (by trend in *IDH2* R172). For patients with *IDH2* R172 mutations, higher median 2-HG concentrations at baseline were significantly associated with a subsequent response. Furthermore, in *IDH2* R172 mutated patients, 2-HG reductions were significantly greater in responders compared to non-responders, while in patients with *IDH2* R140 mutations, 2-HG levels were suppressed from baseline levels irrespective of clinical response category [60].

When looking at co-mutations at baseline, responding patients had significantly fewer baseline mutations compared with non-responding patients. *FLT3*-ITD and -TKD mutations as well as *NRAS* mutations were associated with primary resistance. Moreover, patients with mutations associated with splicing were less likely to respond to enasidenib (Table 2) [60].

Similar observations were made by Amatangelo et al. [59], who investigated predictors of response/resistance in 125 r/r AML patients treated with enasidenib. The authors found that IDH2-inhibition was associated with potent reduction of 2-HG levels irrespective of response in both, R172- and R140-mutated patients. Moreover, clinical responses to IDH2-inhibition did not correlate with *IDH2* allele burden. As expected from preclinical data, the authors could show that clinical response was paralleled by induction of myeloid differentiation. On a genetic level, patients who achieved a response had significantly fewer co-occurring mutations than did non-responders. Analysis of co-mutational status revealed a significantly lower ORR in patients with co-occurring *NRAS* mutations (Table 2). The overall mutational burden was higher in patients with *NRAS* (G12, G13, or Q61) mutations, and *NRAS* mutations were frequently found to be subclonal [59].

Quek et al. examined mechanisms of secondary resistance to enasidenib in 37 patients via paired whole-exome or targeted sequencing, karyotyping and measurement of 2-HG levels at treatment initiation and relapse [62]. The authors found that enasidenib therapy restored differentiation either from ancestral or terminal AML clones and, rarely, wildtype hematopoiesis. In most patients, 2-HG remained suppressed at relapse, suggesting that relapse clones were not dependent on mutated *IDH2*. The mechanisms of secondary resistance were diverse: relapse arose by the clonal evolution or selection of terminal or ancestral clones. Specifically, clonal selection of del(7q), newly acquired *IDH1* mutations (isoform switching), as well as mutations in *FLT3*, *CBL* and *RUNX1* were detected at relapse (Table 2). Of note, no second site mutations in *IDH2* and no acquired *RAS* mutations occurred in this study. In contrast, Intlekofer et al. describe *IDH2* second site mutations acquired *in trans* as a mechanism of secondary resistance to enasidenib, leading to restored 2-HG production (Table 2) [61].

A recent work by Wang et al. further revealed mechanisms of clinical resistance to IDH inhibition. Combining DNA sequencing, RNA sequencing and cytosine methylation profiling in serial samples from 60 *IDH1*- or *IDH2*-mutant AML patients treated with an IDH inhibitor, they showed leukemia stemness to be a major driver of primary resistance to IDH inhibition. Clonal selection of mutations in *RUNX1/CEBPA* or RTK pathway genes was the main driver of acquired resistance to IDH inhibition, along with BCL6 corepressor (*BCOR*) and *TET2* mutations (Table 2) [63]. While TET2 is directly affected by the 2-HG mediated oncometabolism (Figure 1), it is not yet clear how loss-of-function mutations in *BCOR*, a transcriptional corepressor [65,66] contribute to acquired resistance to IDH inhibition.

## 3. Hypomethylating Agents (HMAs)

In AML patients not eligible for IC or allogeneic HSCT, monotherapy with hypomethylating agents has been frequently used in the past. The pyrimidine analogs 5-azacitidine (5-AZA) and decitabine both target DNA methyltransferases which induce epigenetic post-mitotic DNA modifications through methylation — a process involving the addition of a methyl group to CpG-enriched regions (Figure 1) [67]. These regions are often located in areas relevant for gene transcription. Changes in methylation patterns, especially in tumor suppressor or oncogenes, are common findings in malignant cells and have been related to leukemogenesis [68,69,70]. Of note, due to its significant outcome benefit for OS, the combination of 5-AZA and VEN (see below) is the new standard of care in the treatment of frail treatment-naïve AML patients [71], further reducing the relevance of HMA as single-agent therapy in AML treatment. However, in the context of maintenance therapy, oral azacytidine was introduced and recently approved by FDA and EMA in patients with AML in first remission after induction chemotherapy who were ineligible for allogeneic HSCT due to superior OS and RFS, compared to placebo in a randomized phase III trial [72].

### Genetic Predictors of Response or Resistance to HMA as Monotherapy

Consistent with the pharmacological mechanism of HMA, several studies have found the presence of mutations in genes associated with epigenetic DNA modification to be a predictor of favorable outcome in patients treated with HMA only.

A retrospective analysis on 42 AML patients receiving either decitabine or 5-AZA suggested an improvement in overall HMA-treatment response for patients with *IDH* mutations with 71.4% versus 22.9% for *IDH* wt patients [73]. Accordingly, superior ORR could also be observed for patients with AML or MDS harboring a *TET2* mutation treated with 5-AZA, independent of their cytogenetic risk status. The protein encoded by *TET2* plays a crucial role in the DNA demethylation processes [74]. Another common epigenetic modifier known to be recurrently mutated in roughly 20% of all AML cases is *DNMT3A* [75,76]. Metzeler et al. observed a significantly higher CR rate in *DNMT3A* mutated cases compared to *DNMT3A* wt in 46 elderly AML patients treated with decitabine. The response rates in patients with concomitant *DNMT3A* and *NPM1* mutations were even better, although present in only a limited number of patients (*n* = 5). Interestingly, unlike previous reports, the group did not detect an association of pretreatment *TET2* or *IDH1/2* mutational status with decitabine response [77].

In a study by Welch et al. investigating the role of mutations and response to decitabine treatment, the presence of a *TP53* mutation was surprisingly associated with blast clearance, while mutations in *RUNX1* and *SRSF2* were associated with non-response [78]. Another study from 2018 confirmed that patients harboring not only *TP53* but also *NRAS* mutations seemed to particularly benefit from treatment with 5-AZA regarding OS compared to IC, LDAC or BSC [79].

## 4. Other Molecular Targets

### 4.1. BCL-2/Venetoclax

The B-cell lymphoma-2 (BCL-2) family of proteins consists of pro-apoptotic and anti-apoptotic members sharing at least one of four BCL-2 homology domains (BH1–4) and is involved in the regulatory network of apoptosis through mitochondrial outer membrane permeabilization and cytochrome c release. The BCL-2 family is divided into three subfamilies: anti-apoptotic members (e.g., BCL-2, BCL-XL, MCL1) inhibit apoptosis by conserving the integrity of the mitochondrial membrane. They prevent the dimerization of BAX and BAK, another subfamily, which are pro-apoptotic members and resemble BCL-2 in the sequence of their homology domains. Their dimerization leads to the release of cytochrome c into the cytosol (Figure 1). The third subfamily comprises the so-called pro-apoptotic BH3-only proteins (BIK, BAD, BID, BIM, BMF, HRK, NOXA and PUMA). They can either directly activate BAX and/or BAK or regulate the antiapoptotic proteins, as reviewed for example in [80]. It has long been recognized that overexpression of BCL-2 is an important driver of resistance to cell death in AML [81] and occurs in 80–90% of AML patient samples [82], rendering BCL-2 an attractive therapeutic target in the treatment of AML.

Venetoclax (ABT-199, VEN) is an orally available BH3-mimetic, selectively binding to BCL-2 and thereby releasing pro-apoptotic proteins such as BAX and BAK (Figure 1). Being successfully employed in the treatment of B-cell lymphomas, it also showed promising in vitro activity against AML cells [83,84]. In clinical studies, VEN showed limited efficacy as monotherapy in patients with r/r AML [85], with response rates of 19%. In contrast, in combination with low-dose cytarabine (LDAC) or HMA, it showed remarkable efficacy in two phase Ib/II studies in untreated AML leading to durable remissions in elderly patients unfit for intensive therapy [9,86]. These results were recently supported by two large phase III studies (VIALE-A and VIALE-C) [71,87], which led to the approval of the two combination therapies by the FDA and EMA for patients older than 75 years or patients unfit for IC. Data from preclinical models suggest that exposure to 5-AZA leads to the induction of a pro-apoptotic protein NOXA, priming AML cells for programmed cell death with VEN, as one mechanism of this clinically observed synergy [88]. The addition of VEN to various IC protocols and/or targeted therapies is currently the subject of various clinical trials (e.g., IC: AC-TRN12616000445471; CAVEAT [89], NCT03214562, NCT03629171).

#### Genetic Predictors of Response or Resistance to Venetoclax

*IDH1/2 mutations*: In an early phase II study with 32 patients with r/r AML receiving VEN monotherapy, the authors noted that the group of *IDH1/2*-mutated patients showed a higher ORR [85]. In the same cohort, Chyla et al. interrogated mechanisms of resistance by analyzing paired bone marrow specimens before treatment and at progress/relapse [90]. The data showed higher rates of blast reduction in patients with *IDH1/2* mutations and/or spliceosome mutations (in particular *SRSF2*). These observations are in line with the results from Chua et al., who investigated VEN in combination with modified IC in a phase Ib study of 51 elderly patients. After a seven-day VEN monotherapy prephase, patients with *NPM1, IDH2, SRSF2* mutated AML showed a higher rate of blast reduction [89]. DiNardo et al. analyzed patterns of response under VEN-based treatment in 81 patients from phase I/II studies. The strongest molecular associations with response were noted in patients with *NPM1*, *IDH1* and *IDH2* mutations, with CR/CRi rates exceeding 80%, in particular in *NPM1* and *IDH2* mutated patients [91] Similar results were reported in larger clinical studies of VEN + HMA in newly diagnosed and r/r AML patients, including VIALE-A, the large phase III trial [71], in which *IDH*-mutated patients consistently reached high response rates leading to long lasting remissions and improved OS (Table 3) [86,87,91,92,93,94]. Sensitivity of *IDH*-mutated AML to VEN has also been noted in vitro in a study with 207 AML specimens from the Leucegene cohort [95]. Chan et al. performed large-scale RNA interference screen and identified BCL-2 to be synthetic lethal to the *IDH1* R132 mutation [96]. As *IDH* mutations themselves represent a therapeutic target, the combination of IDH inhibitors with VEN is currently being investigated in clinical trials (NCT04092179, NCT03471260).

*NPM1 mutations*: *Nucleophosmin-1* mutations (*NPM1*) occur in almost a third of AML patients and are typically associated with favorable prognosis in patients treated with standard IC or HMA. Current data strongly suggest that *NPM1*-mutated AML also shows a favorable response to VEN-based therapy. In a cohort of 86 patients with r/r AML retrospectively analyzed by Stahl and colleagues, *NPM1* mutations predicted higher response rates to VEN-based combination therapies [100] in line with results from DiNardo et al. [91]. Consistent results from several clinical cohorts comprising newly diagnosed as well as r/r AML patients were reported by other groups, underlining the remarkable response rates and prolonged OS under VEN-based therapy to VEN-based therapy in *NPM1*-mutated AML [87,89,92,97,100], as summarized in Table 3. In a small retrospective study, Lachowiez et al. compared VEN + HMA to HMA monotherapy as well as conventional IC in patients of age > 65 with *NPM1*-mutated AML. The VEN + HMA group showed significantly improved OS compared to both HMA monotherapy and IC [97]. The extraordinary sensitivity of *NPM1*-mutated AML to VEN is also supported by *in vitro* studies (Table 3) [95].

*Spliceosome mutations*: In contrast, the prognostic impact of mutations in genes associated with the spliceosome (*SRSF2, SF3B1, ZRSR2, U2AF1*) with respect to VEN therapy is inconsistent. *SRSF2* mutations seem to frequently co-occur with *IDH2* mutations [59], conferring a favorable prognosis under VEN-based therapy [92]. However, a recent study by Lachowiez et al. compared outcomes between patients with and without spliceosome mutations under VEN-based therapy. Responses and median OS were similar for mutated spliceosome vs. wt patients. Nevertheless, specific co-mutation pairs seemed to impact the outcome: *IDH2* mutations were more frequent in patients with *SRSF2* mutations and showed favorable outcomes (1- and 2-year OS of 100% and 88%) as opposed to *RAS* mutations, which were enriched in patients with *U2AF1* mutations and were associated with worse OS (median OS, 8 months) [98]. For *SF3B1* mutations, current clinical data suggest lower response rates [102] and worse OS in patients with r/r AML under VEN-based therapy (Table 3) [100].

*TP53 mutations*: *TP53* mutations in AML are often associated with a complex karyotype and with adverse prognosis [104]. Current data suggests that VEN does not improve the dismal outcome of *TP53*-mutated AML. DiNardo et al. reported *TP53* mutations and *RUNX1* mutations to be significantly associated with non-responsiveness to VEN-based therapy. Likewise, secondary resistance resulted from the selection of clones with likely biallelic perturbation of *TP53* [91]. This competitive outgrowth of *TP53*-mutated clones in patients was corroborated with in vitro data, in which CRISPR/CAS9 modified *TP53*-deficient cells showed a selective advantage under therapeutic pressure with 5-AZA, VEN and 5-AZA + VEN [91], consistent with data from other in vitro studies [95,103,105]. Aiming to identify knockouts leading to VEN resistance, Nechiporuk et al. performed a genome-wide CRISPR/CAS9 screen and found inactivation of TP53, BAX, and PMAIP1 to be potent drivers of VEN resistance, resulting from decreased BCL-2 expression and/or reliance on alternative BCL-2 family members such as BCL2L1 [105]. Consistently, in all subsequent clinical studies, patients with *TP53*-mutated AML did not demonstrate any objective response to VEN-based therapy and had worse OS [87,92,99,100]. Neither the large phase III trial VIALE-A [71] nor two recent smaller studies [106,107] could show a significant benefit of adding VEN to HMA in *TP53*-mutated AML (Table 3). In summary, VEN does not seem to improve the dismal outcome of patients with *TP53*-mutated AML and other therapeutic approaches are needed to address this aggressive subgroup of AML.

Activating kinase mutations/*FLT3 mutations*: *FLT3* is the most frequently mutated gene in AML [5], while other members of the RAS-RTK pathway such as *CBL, KIT, NRAS/KRAS, PTPN11* are less frequent and their impact on treatment outcomes is less clear. Early data on VEN monotherapy suggested primary resistance in patients with *FLT3*-ITD or *PTPN11* mutations as well as outgrowth of clones with *FLT3/PTPN11* mutations under VEN therapy, hinting at a role of RAS-RTK pathway mutations in VEN resistance [85,90]. Likewise, a strong association of activating kinase mutations (*FLT3, CBL, KIT, NRAS/KRAS*) with non-response and lower OS under VEN + HMA or VEN + LDAC was reported by multiple other groups [86,87,91,92,93,97,98,101], as summarized in Table 3. Again, secondary resistance resulted from progressive expansion of clones with activated kinases, particularly *FLT3*-ITD [91]. In vitro sensitivity analyses suggest reliance on other anti-apoptotic proteins such as MCL1 and BCL-XL in AML with mutations in the RAS-RTK pathway as one mechanism of resistance [95,102]. Preclinical models of VEN in combination with FLT3-inhibition have shown promising synergy via downregulation of MCL1 and BCL-XL in preclinical models [108,109,110]. Early clinical studies investigating doublet combinations (NCT03625505: VEN + gilteritinib) or triplet combinations (NCT03661307: VEN + decitabine + quizartinib, NCT04140487: VEN + 5-AZA + gilteritinib) are ongoing and show promising first results [111]. Other ways of overcoming VEN resistance include combinations with MCL-1 inhibitors [112,113,114,115,116] or concomitant targeting of the BCL2 and MAPK pathways by VEN and the MEK1/2 inhibitor cobimetinib [117].

Other predictors—FAB classification: Interestingly, there seems to be an association between French–American–British (FAB) classification and VEN resistance. As observed by Pei et al. in 100 newly diagnosed, previously untreated AML patients treated with VEN + 5-AZA, non-response was associated with the presence of RAS-RTK pathway mutations (Table 3), and was even stronger with FAB-M5 maturation state [101]. Mechanistically, the authors demonstrated that monocytic AML loses expression of BCL-2 and is preferentially reliant on MCL1 instead. In serial samples of relapsed patients, the authors could show that treatment with VEN + 5-AZA selects monocytically differentiated clones which are often present as subclones at the initiation of treatment [101]. Similar observations were made in the Leucegene cohort, in which resistance was seen in the FAB M5B class, whereas the FAB M1 class was associated with sensitivity [95]. In the BEAT AML cohort, FAB M4 and M5 were significantly associated with resistance, while M3 and *PML-RARA* translocations were predictive for sensitivity [102].

Several other concepts of VEN resistance beyond the acquisition of somatic mutations have emerged in the last few years. Among them, extracellular vesicles [118,119,120], upregulation of fatty acid oxidation [121] and reduced mitochondrial priming [116] have been shown to be important mediators of resistance to BCL-2 inhibition.

### 4.2. Glasdegib/Inhibition of the Hedgehog Signaling Pathway

The hedgehog pathway is extremely conserved and plays a pivotal role, for example in embryogenesis. Dysregulation of the hedgehog pathway is described in many ma-lignancies including AML (reviewed in [122]) where it has been associated with persistence and expansion of leukemic stem cells and resistance to chemotherapy [123,124]. Glasdegib is a selective inhibitor of smoothened (SMO), a cellular receptor and key regulator of the hedgehog pathway [124]. Glasdegib in combination with LDAC showed a significant improvement in OS compared to LDAC alone (8.8 vs. 4.9 months) in the randomized phase II BRIGHT AML 1003 trial [12] and has subsequently been approved by FDA and EMA for AML patients ≥ 75 years or those who are ineligible for IC due to comorbidities. Interestingly, survival benefit was not restricted to patients receiving CR [125]. No genetic predictors for response to glasdegib were identified so far; however, the presence of a *DNMT3A* mutation seems to negatively impact OS in patients with secondary AML and glasdegib + LDAC treatment [126].

## 5. Antigen-Targeting

### CD33/Gemtuzumab Ozogamicin

Due to its high surface expression on AML cells and lack of expression on early hematopoietic stem cells, the transmembrane antigen CD33 has been recognized as an ideal target for antibody-based therapy in AML. The antibody drug conjugate gemtuzumab ozogamicin (GO) is a humanized IgG4 anti-CD33 monoclonal antibody linked to a calicheamicin derivate, a potent antitumor antibiotic known for its strong DNA damaging effects mostly through DNA strand breaks and activation of the mitochondrial apoptosis pathway via caspases 3 and 9 [127,128].

GO was approved in 2017 by the FDA and in 2018 by the EMA in addition to standard chemotherapy with daunorubicin and cytarabine in patients with de novo CD33-positive acute myeloid leukemia with additional approval in the r/r setting as monotherapy by the FDA [129]. In the ALFA-0701 phase III clinical trial [11,130], patients in the GO arm showed a significant improvement in event-free survival (EFS) and had a lower relapse risk compared to patients in the control arm. The CD33-expression level on AML blasts did not seem to influence the positive impact of GO on EFS in the ALFA-0701 trial, an observation that was also made in another clinical study [10], but remains a contradictory finding possibly due to diverse dosing study regimens and age differences in the study populations [131]. Interestingly, GO seems to particularly improve outcomes in patients with core-binding factor (CBF) leukemia, as shown in a subgroup analysis focusing on defined ELN risk groups [10].

A recent retrospective analysis aimed at evaluating possible genetic predictors for GO response. To this end, 230 patients of the ALFA-0701 study cohort were genetically characterized. The greatest benefit of GO treatment on EFS was observed in patients harboring recurrent mutations in *NPM1, FLT3*-ITD, *FLT3*-TKD, *NRAS, KRAS* and *SRSF2*. In contrast, in patients with *RUNX1, ASXL1* and *TP53* mutations, EFS was not improved by the addition of GO [132]. Interestingly, *NRAS* and *KRAS* mutations were associated with GO response, a finding of potential clinical relevance as these RTK pathways mutations have been consistently reported as poor prognostic markers in various treatment approaches, including FLT3 or IDH1/2 inhibitors as summarized above.

## 6. Novel Chemotherapeutic Agents

### CPX-351

CPX-351 is a liposomal dual drug containing cytarabine and daunorubicin at a fixed 5:1 ratio encapsulated in bi-layered liposomes [133]. This formulation ensures longer-lasting plasma concentrations with detectable drug levels at >7 days post-application [133,134]. CPX-351 was approved by the FDA and EMA for the treatment of adults with newly diagnosed AML with myelodysplasia-related changes (AML-MRC) and therapy-associated AML (t-AML) [135,136]. These two sub-entities are associated with dismal prognosis in most cases [137].

Molecular data for the identification of molecular predictors of response are present from various studies. Interestingly, mutations in *ASXL1* and *RUNX1* were not associated with poorer responses [138] or OS rates in several studies [139] despite the fact that their presence leads to classification into the adverse risk group according to ELN classification [1]. Data regarding *TP53* mutations were less clear with some studies showing no difference in response or OS [139,140], while others reported an impaired clinical outcome [138]. Complex cytogenetics, a parameter likewise defining the ELN adverse-risk group [1], was associated with poorer outcome in two studies [139,140]. One study reports that the presence of spliceosome mutations is associated with a better OS [138]. In summary, future studies will probably disentangle the impact of classical molecular risk parameters in the treatment with CPX-351. In addition, studies exploring the impact of CPX-351 in other subgroups of AML are under way, most probably providing more data on molecular predictors beyond the above-mentioned high-risk genes.

## 7. Discussion

After many years with limited therapeutic progress, AML treatment has greatly improved since 2017. Individual risk stratification by molecular profiling of leukemia samples and availability of many effective agents for molecular subtypes, e.g., FLT3 inhibitors or IDH1/2 inhibitors, allow the first steps towards personalized AML therapy. Nevertheless, great therapeutic obstacles remain, as demonstrated by the large number of AML patients who experience refractory disease or leukemia relapse.

As highlighted in the sections above, mutations that remain difficult to target but are associated with adverse prognosis are particularly challenging in the context of personalized therapy. Besides continuous efforts to identify means to specifically overcome the dismal effects of these mutations, these circumstances highlight the necessity of comprehensive molecular characterization in clinical trials, as individual substance–mutation interactions might reveal unexpected effects on response and prognosis. Though pathophysiologically not always fully understood, these data could likewise help us to design personalized therapeutic concepts.

Across almost all conventional and targeted therapies, mutations in the RAS-RAF-MEK-ERK pathway and *TP53* appear particularly problematic. Therefore, many efforts are currently being made to improve the dismal prognosis of patients with these molecular aberrations.

With regard to *TP53*-mutated/deleted AML, various agents in clinical testing give reason for optimism. Magrolimab is an IgG4 anti-CD47 monoclonal antibody targeting the “don’t eat me” surface molecule on cells, unleashing the macrophage immune checkpoint and leading to phagocytosis. CD47 is often expressed in high levels on AML cells, making it a target of interest. Of note, various clinical trials have reported high CR/CRi rates of *TP53*- mutated high-risk MDS and AML. Likewise, the studies report high rates of MRD negativity in magrolimab-treated *TP53*-mutated patients. Magrolimab is currently subject to further clinical trials. In addition, more anti-CD47 monoclonal antibodies are currently in development and/or clinical testing (reviewed in [141]).

Other promising agents in *TP53*-mutated/deleted AML are PRIMA-1 (p53-Reactivation and Induction of Massive Apoptosis-1) and its clinically advanced analogue eprenetapopt (PRIMA-1met/APR-246). PRIMA-1 and eprenetapopt are pro-drugs that restore *TP53* wildtype transcriptional function through the biologically active compound methylene quinuclidinone (MQ) [142,143]. In combination with 5-AZA, eprenetapopt was well tolerated and led to high response rates in two phase II clinical trials [144,145]. The clinical role of eprenetapopt and possible combination treatments are currently being investigated in a series of trials.

As outlined in detail in this review, mutations affecting the RAS-RAF-MEK-ERK pathway are frequently associated with primary and secondary resistance to various otherwise highly effective agents. Overcoming this resistance is, however, challenging. Promising preclinical results exist for various agents inhibiting pathway components. For example, in the pre-clinical setting, the pan-RAF inhibitor LY3009120 showed pro-apoptotic and anti-proliferative effects in *FLT3-* and *RAS*-mutated AML cells [146] and effects in AML cells were even enhanced when combining LY3009120 with a BCL-2 inhibitor [147] or sorafenib [146]. Likewise, MEK inhibitors in various combinations showed promising results in pre-clinical testing [148,149]. These encouraging data are in contrast to the rather disappointing results of early clinical trials. For example, the selective MEK inhibitor selumetinib showed only modest single-agent activity in r/r AML or untreated elderly AML patients [150]. Likewise, combined MEK and AKT inhibition showed no clinical effect in *RAS*-mutated AML [151]. Though comprehensive molecular data explaining the rather low efficacy of these drugs are still lacking, clonal diversity, genetic complexity, and alterations in the proteome/metabolome will most certainly account at least in part for these as yet unsatisfactory results. Of note, GO seems to be an exemption, as activating signaling mutations, including mutations in *NRAS* and *KRAS* in favorable and intermediate ELN 2017 risk AML, are associated with better EFS, most probably explained by increased expression of CD33 on AML blasts [132].

Development of new substances for AML therapy is steadily moving forward, and agents like menin inhibitors for MLL-rearranged leukemia and the anti-TIM-3 antibody sabatolimab are promising candidates [152,153]. However, for substances in preclinical and clinical testing, large-scale, in-depth analyses regarding genetic predictors are still pending. In addition, other molecular predictors beyond genomic characteristics will most probably be of relevance, in particular with regard to immune checkpoint inhibitors.

Techniques for deep and comprehensive molecular analysis are developing at a rapid pace. In particular, single cell analyses on one, two or even multiple levels (genome, chromatin, transcriptome, proteome, mitochondrial DNA) allow unpreceded insights into molecular processes [154,155,156,157,158,159] and thus provide data better mirroring the biologic complexity of physiologic and pathologic processes. Though the costs and complexity of data analysis currently prevent implementation of these techniques on a large scale or in routine diagnostics, it appears as a matter of time until they will broadly be used to enhance our understanding of response, relapse, and resistance in the field of hemato-oncology.

## 8. Conclusions

Our understanding of the molecular background of AML is proceeding with ravishing speed, revealing a multitude of recurrent mutations with impact on response to specific therapies and prognosis. Some mutations have led to the development of specific inhibitors-and hopefully, there are more to come. However, the major part of genetic changes cannot yet be targeted directly, but rather serves as predictor of response. Comprehensive molecular profiling has become integral part of clinical studies in the AML setting, allowing the identification of molecular subtypes that benefit the most or the least from a specific therapy. In summary, this ongoing development paves the way to truly individualized AML therapy, leading to the legitimate hope that personalized treatments can soon be offered to every AML patient.

## Figures and Tables

**Table 1:** Mutations associated with response or resistance to FLT3 inhibition.

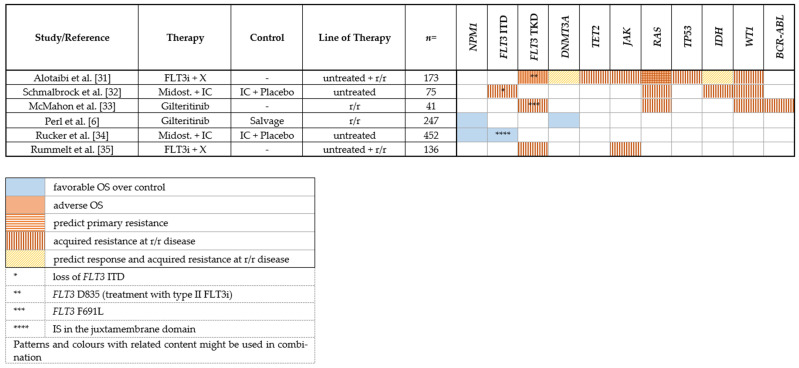

**Table 2:** Mutations associated with response or resistance to IDH1/2 inhibition.

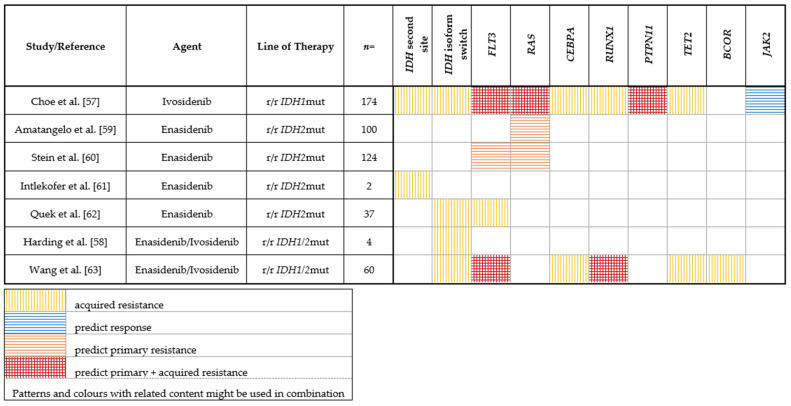

**Table 3:** Mutations associated with response or resistance to venetoclax.

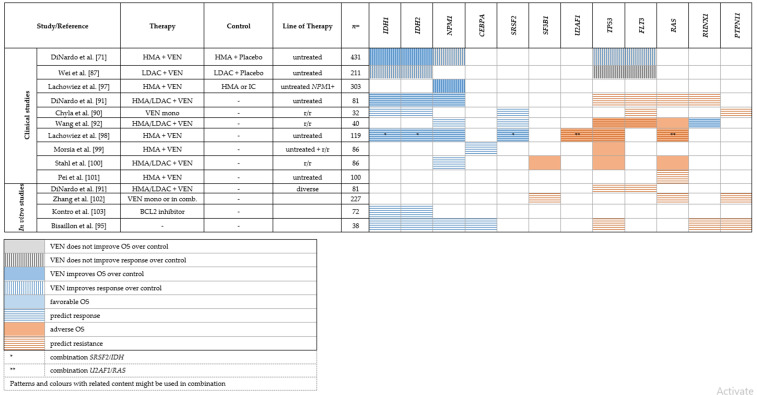

## References

[1] Döhner H., Estey E., Grimwade D., Amadori S., Appelbaum F.R., Büchner T., Dombret H., Ebert B.L., Fenaux P., Larson R.A. (2017). Diagnosis and management of AML in adults: 2017 ELN recommendations from an international expert panel. Blood.

[2] The Cancer Genome Atlas Research Network (2013). Genomic and epigenomic landscapes of adult de novo acute myeloid leukemia. N. Engl. J. Med..

[3] Papaemmanuil E., Gerstung M., Bullinger L., Gaidzik V.I., Paschka P., Roberts N.D., Potter N.E., Heuser M., Thol F., Bolli N. (2016). Genomic Classification and Prognosis in Acute Myeloid Leukemia. N. Engl. J. Med..

[4] Tyner J.W., Tognon C.E., Bottomly D., Wilmot B., Kurtz S.E., Savage S.L., Long N., Schultz A.R., Traer E., Abel M. (2018). Functional genomic landscape of acute myeloid leukaemia. Nat. Cell Biol..

[5] Stone R.M., Mandrekar S.J., Sanford B.L., Laumann K., Geyer S., Bloomfield C.D., Thiede C., Prior T.W., Döhner K., Marcucci G. (2017). Midostaurin plus Chemotherapy for Acute Myeloid Leukemia with a *FLT3* Mutation. N. Engl. J. Med..

[6] Perl A.E., Martinelli G., Cortes J.E., Neubauer A., Berman E., Paolini S., Montesinos P., Baer M.R., Larson R.A., Ustun C. (2019). Gilteritinib or Chemotherapy for Relapsed or Refractory FLT3-Mutated AML. N. Engl. J. Med..

[7] Dinardo C.D., Stein E.M., DE Botton S., Roboz G.J., Altman J.K., Mims A.S., Swords R., Collins R.H., Mannis G.N., Pollyea D.A. (2018). Durable Remissions with Ivosidenib inIDH1-Mutated Relapsed or Refractory AML. N. Engl. J. Med..

[8] Stein E.M., Dinardo C.D., Pollyea D.A., Fathi A.T., Roboz G.J., Altman J.K., Stone R.M., DeAngelo D.J., Levine R.L., Flinn I.W. (2018). Enasidenib in mutant IDH2 relapsed or refractory acute myeloid leukemia. Blood.

[9] DiNardo C.D., Pratz K.W., Letai A., Jonas B., Wei A.H., Thirman M., Arellano M., Frattini M.G., Kantarjian H., Popovic R. (2018). Safety and preliminary efficacy of venetoclax with decitabine or azacitidine in elderly patients with previously untreated acute myeloid leukaemia: A non-randomised, open-label, phase 1b study. Lancet Oncol..

[10] Burnett A.K., Hills R.K., Milligan D., Kjeldsen L., Kell J., Russell N.H., Yin J.A., Hunter A., Goldstone A.H., Wheatley K. (2011). Identification of Patients With Acute Myeloblastic Leukemia Who Benefit From the Addition of Gemtuzumab Ozogamicin: Results of the MRC AML15 Trial. J. Clin. Oncol..

[11] Castaigne S., Pautas C., Terré C., Raffoux E., Bordessoule D., Bastie J.-N., Legrand O., Thomas X., Turlure P., Reman O. (2012). Effect of gemtuzumab ozogamicin on survival of adult patients with de-novo acute myeloid leukaemia (ALFA-0701): A randomised, open-label, phase 3 study. Lancet.

[12] Cortes J.E., Heidel F.H., Hellmann A., Fiedler W., Smith B.D., Robak T., Montesinos P., Pollyea D.A., DesJardins P., Ottmann O. (2018). Randomized comparison of low dose cytarabine with or without glasdegib in patients with newly diagnosed acute myeloid leukemia or high-risk myelodysplastic syndrome. Leukemia.

[13] Rosnet O., Buhring H.J., Marchetto S., Rappold I., Lavagna C., Sainty D., Arnoulet C., Chabannon C., Kanz L., Hannum C. (1996). Human FLT3/FLK2 receptor tyrosine kinase is expressed at the surface of normal and malignant hematopoietic cells. Leukemia.

[14] Takahashi S. (2011). Downstream molecular pathways of FLT3 in the pathogenesis of acute myeloid leukemia: Biology and therapeutic implications. J. Hematol. Oncol..

[15] Stirewalt D.L., Radich J.P. (2003). The role of FLT3 in haematopoietic malignancies. Nat. Cancer.

[16] Nagel G., Weber D., Fromm E., Erhardt S., Lübbert M., Fiedler W., Kindler T., Krauter J., Brossart P., German-Austrian AML Study Group (AMLSG) (2017). Epidemiological, genetic, and clinical characterization by age of newly diagnosed acute myeloid leukemia based on an academic population-based registry study (AMLSG BiO). Ann. Hematol..

[17] Herold T., Rothenberg-Thurley M., Grunwald V.V., Janke H., Goerlich D., Sauerland M.C., Konstandin N.P., Dufour A., Schneider S., Neusser M. (2020). Validation and refinement of the revised 2017 European LeukemiaNet genetic risk stratification of acute myeloid leukemia. Leukemia.

[18] Gale R.E., Green C., Allen C., Mead A.J., Burnett A.K., Hills R.K., Linch D.C. (2008). The impact of FLT3 internal tandem duplication mutant level, number, size, and interaction with NPM1 mutations in a large cohort of young adult patients with acute myeloid leukemia. Blood.

[19] Daver N., Cortes J., Ravandi F., Patel K.P., Burger J.A., Konopleva M., Kantarjian H. (2015). Secondary mutations as mediators of resistance to targeted therapy in leukemia. Blood.

[20] Smith C.C., Wang Q., Chin C.-S., Salerno S., Damon L.E., Levis M.J., Perl A.E., Travers K.J., Wang S., Hunt J.P. (2012). Validation of ITD mutations in FLT3 as a therapeutic target in human acute myeloid leukaemia. Nature.

[21] Propper D.J., McDonald A.C., Man A., Thavasu P., Balkwill F., Braybrooke J.P., Caponigro F., Graf P., Dutreix C., Blackie R. (2001). Phase I and Pharmacokinetic Study of PKC412, an Inhibitor of Protein Kinase C. J. Clin. Oncol..

[22] Fabbro D., Buchdunger E., Wood J., Mestan J., Hofmann F., Ferrari S., Mett H., O’Reilly T., Meyer T. (1999). Inhibitors of Protein KinasesCGP 41251, a Protein Kinase Inhibitor with Potential as an Anticancer Agent. Pharmacol. Ther..

[23] Ke Y.-Y., Singh V.K., Coumar M., Hsu Y.C., Wang W.-C., Song J.-S., Chen C.-H., Lin W.-H., Wu S.-H., Hsu J.T.A. (2015). Homology modeling of DFG-in FMS-like tyrosine kinase 3 (FLT3) and structure-based virtual screening for inhibitor identification. Sci. Rep..

[24] Stone R.M., DeAngelo D.J., Klimek V., Galinsky I., Estey E., Nimer S.D., Grandin W., Lebwohl D., Wang Y., Cohen P. (2005). Patients with acute myeloid leukemia and an activating mutation in FLT3 respond to a small-molecule FLT3 tyrosine kinase inhibitor, PKC412. Blood.

[25] Mori M., Kaneko N., Ueno Y., Yamada M., Tanaka R., Saito R., Shimada I., Mori K., Kuromitsu S. (2017). Gilteritinib, a FLT3/AXL inhibitor, shows antileukemic activity in mouse models of FLT3 mutated acute myeloid leukemia. Investig. New Drugs.

[26] Perl A.E., Altman J.K., Cortes J., Smith C., Litzow M., Baer M.R., Claxton D., Erba H.P., Gill S., Goldberg S. (2017). Selective inhibition of FLT3 by gilteritinib in relapsed or refractory acute myeloid leukaemia: A multicentre, first-in-human, open-label, phase 1–2 study. Lancet Oncol..

[27] Lee L.Y., Hernandez D., Rajkhowa T., Smith S.C., Raman J.R., Nguyen B., Small D., Levis M. (2017). Preclinical studies of gilteritinib, a next-generation FLT3 inhibitor. Blood.

[28] Auclair D., Miller D., Yatsula V., Pickett W., Carter C., Chang Y., Zhang X., Wilkie D., Burd A., Shi H. (2007). Antitumor activity of sorafenib in FLT3-driven leukemic cells. Leukemia.

[29] Burchert A., Bug G., Fritz L.V., Finke J., Stelljes M., Röllig C., Wollmer E., Wäsch R., Bornhäuser M., Berg T. (2020). Sorafenib Maintenance After Allogeneic Hematopoietic Stem Cell Transplantation for Acute Myeloid Leukemia With FLT3–Internal Tandem Duplication Mutation (SORMAIN). J. Clin. Oncol..

[30] Xuan L., Wang Y., Huang F., Fan Z., Xu Y., Sun J., Xu N., Deng L., Li X., Liang X. (2020). Sorafenib maintenance in patients with FLT3-ITD acute myeloid leukaemia undergoing allogeneic haematopoietic stem-cell transplantation: An open-label, multicentre, randomised phase 3 trial. Lancet Oncol..

[31] Alotaibi A.S., Yilmaz M., Kanagal-Shamanna R., Loghavi S., Kadia T.M., DiNardo C.D., Borthakur G., Konopleva M., Pierce S.A., Wang S.A. (2020). Patterns of Resistance Differ in Patients with Acute Myeloid Leukemia Treated with Type I versus Type II FLT3 Inhibitors. Blood Cancer Discov..

[32] Schmalbrock L.K., Dolnik A., Cocciardi S., Sträng E., Theis F., Jahn N., Panina E., Blätte T.J., Herzig J., Skambraks S. (2021). Clonal evolution of acute myeloid leukemia with FLT3-ITD mutation under treatment with midostaurin. Blood.

[33] McMahon C.M., Ferng T., Canaani J., Wang E.S., Morrissette J.J., Eastburn D.J., Pellegrino M., Durruthy-Durruthy R., Watt C.D., Asthana S. (2019). Clonal Selection with RAS Pathway Activation Mediates Secondary Clinical Resistance to Selective FLT3 Inhibition in Acute Myeloid Leukemia. Cancer Discov..

[34] Rücker F.G., Du L., Luck T.J., Benner A., Krzykalla J., Gathmann I., Voso M.T., Amadori S., Prior T.W., Brandwein J.M. (2021). Molecular landscape and prognostic impact of FLT3-ITD insertion site in acute myeloid leukemia: RATIFY study results. Leukemia.

[35] Rummelt C., Gorantla S.P., Meggendorfer M., Charlet A., Endres C., Döhner K., Heidel F.H., Fischer T., Haferlach T., Duyster J. (2020). Activating JAK-mutations confer resistance to FLT3 kinase inhibitors in FLT3-ITD positive AML in vitro and in vivo. Leukemia.

[36] Chen F., Ishikawa Y., Akashi A., Naoe T., Kiyoi H. (2016). Co-expression of wild-type FLT3 attenuates the inhibitory effect of FLT3 inhibitor on FLT3 mutated leukemia cells. Oncotarget.

[37] Smith C.C., Paguirigan A., Jeschke G.R., Lin K.C., Massi E., Tarver T., Chin C.-S., Asthana S., Olshen A., Travers K.J. (2017). Heterogeneous resistance to quizartinib in acute myeloid leukemia revealed by single-cell analysis. Blood.

[38] Heidel F., Solem F.K., Breitenbuecher F., Lipka D.B., Kasper S., Thiede M.H., Brandts C.H., Serve H., Roesel J., Giles F. (2005). Clinical resistance to the kinase inhibitor PKC412 in acute myeloid leukemia by mutation of Asn-676 in the FLT3 tyrosine kinase domain. Blood.

[39] Baker S.D., Zimmerman E.I., Wang Y.-D., Orwick S., Zatechka D.S., Buaboonnam J., Neale G.A., Olsen S.R., Enemark E.J., Shurtleff S. (2013). Emergence of Polyclonal FLT3 Tyrosine Kinase Domain Mutations during Sequential Therapy with Sorafenib and Sunitinib in FLT3-ITD–Positive Acute Myeloid Leukemia. Clin. Cancer Res..

[40] Mylonas E., Yoshida K., Frick M., Hoyer K., Christen F., Kaeda J., Obenaus M., Noerenberg D., Hennch C., Chan W. (2020). Single-cell analysis based dissection of clonality in myelofibrosis. Nat. Commun..

[41] Agarwal A., A Eide C., Harlow A., Corbin A.S., Mauro M.J., Druker B., Corless C.L., Heinrich M., Deininger M.W. (2008). An activating KRAS mutation in imatinib-resistant chronic myeloid leukemia. Leukemia.

[42] Krauth M.-T., Alpermann T., Bacher U., Eder C., Dicker F., Ulke M., Kuznia S., Nadarajah N., Kern W., Haferlach C. (2014). WT1 mutations are secondary events in AML, show varying frequencies and impact on prognosis between genetic subgroups. Leukemia.

[43] Paschka P., Marcucci G., Ruppert A.S., Whitman S.P., Mrózek K., Maharry K., Langer C., Baldus C.D., Zhao W., Powell B.L. (2008). Wilms’ Tumor 1 Gene Mutations Independently Predict Poor Outcome in Adults With Cytogenetically Normal Acute Myeloid Leukemia: A Cancer and Leukemia Group B Study. J. Clin. Oncol..

[44] Gaidzik V.I., Schlenk R.F., Moschny S., Becker A., Bullinger L., Corbacioglu A., Krauter J., Schlegelberger B., Ganser A., Döhner H. (2009). Prognostic impact of WT1 mutations in cytogenetically normal acute myeloid leukemia: A study of the German-Austrian AML Study Group. Blood.

[45] Damm F., Heuser M., Morgan M., Yun H., Großhennig A., Göhring G., Schlegelberger B., Döhner K., Ottmann O., Lübbert M. (2010). Single Nucleotide Polymorphism in the Mutational Hotspot of WT1 Predicts a Favorable Outcome in Patients With Cytogenetically Normal Acute Myeloid Leukemia. J. Clin. Oncol..

[46] Alotaibi A.S., Yilmaz M., Loghavi S., Dinardo C., Borthakur G., Kadia T.M., Thakral B., Pemmaraju N., Issa G.C., Konopleva M. (2020). Emergence of BCR–ABL1 Fusion in AML Post–FLT3 Inhibitor-Based Therapy: A Potentially Targetable Mechanism of Resistance – A Case Series. Front. Oncol..

[47] Dang L., White D.W., Gross S., Bennett B.D., Bittinger M.A., Driggers E.M., Fantin V.R., Jang H.G., Jin S., Keenan M.C. (2009). Cancer-associated IDH1 mutations produce 2-hydroxyglutarate. Nature.

[48] Figueroa M.E., Abdel-Wahab O., Lu C., Ward P.S., Patel J., Shih A., Li Y., Bhagwat N., VasanthaKumar A., Fernandez H.F. (2010). Leukemic IDH1 and IDH2 Mutations Result in a Hypermethylation Phenotype, Disrupt TET2 Function, and Impair Hematopoietic Differentiation. Cancer Cell.

[49] Lu C., Ward P.S., Kapoor G.S., Rohle D., Turcan S., Abdel-Wahab O., Edwards C.R., Khanin R., Figueroa M.E., Melnick A. (2012). IDH mutation impairs histone demethylation and results in a block to cell differentiation. Nature.

[50] Xu W., Yang H., Liu Y., Yang Y., Wang P., Kim S.-H., Ito S., Yang C., Wang P., Xiao M.-T. (2011). Oncometabolite 2-Hydroxyglutarate Is a Competitive Inhibitor of α-Ketoglutarate-Dependent Dioxygenases. Cancer Cell.

[51] Frick M., Chan W., Arends C.M., Hablesreiter R., Halik A., Heuser M., Michonneau D., Blau O., Hoyer K., Christen F. (2019). Role of Donor Clonal Hematopoiesis in Allogeneic Hematopoietic Stem-Cell Transplantation. J. Clin. Oncol..

[52] Genovese G., Kähler A.K., Handsaker R.E., Lindberg J., Rose S.A., Bakhoum S.F., Chambert K., Mick E., Neale B.M., Fromer M. (2014). Clonal Hematopoiesis and Blood-Cancer Risk Inferred from Blood DNA Sequence. N. Engl. J. Med..

[53] Popovici-Muller J., Lemieux R.M., Artin E., Saunders J.O., Salituro F.G., Travins J., Cianchetta G., Cai Z., Zhou D., Cui D. (2018). Discovery of AG-120 (Ivosidenib): A First-in-Class Mutant IDH1 Inhibitor for the Treatment of IDH1 Mutant Cancers. ACS Med. Chem. Lett..

[54] Wang F., Travins J., DeLaBarre B., Penard-Lacronique V., Schalm S., Hansen E., Straley K., Kernytsky A., Liu W., Gliser C. (2013). Targeted Inhibition of Mutant IDH2 in Leukemia Cells Induces Cellular Differentiation. Science.

[55] Yen K., Travins J., Wang F., David M., Artin E., Straley K., Padyana A., Gross S., DelaBarre B., Tobin E. (2017). AG-221, a First-in-Class Therapy Targeting Acute Myeloid Leukemia Harboring Oncogenic IDH2 Mutations. Cancer Discov..

[56] Roboz G.J., Dinardo C.D., Stein E.M., De Botton S., Mims A.S., Prince G.T., Altman J.K., Arellano M.L., Donnellan W., Erba H.P. (2020). Ivosidenib induces deep durable remissions in patients with newly diagnosed IDH1-mutant acute myeloid leukemia. Blood.

[57] Choe S., Wang H., Dinardo C.D., Stein E.M., De Botton S., Roboz G.J., Altman J.K., Mims A.S., Watts J.M., Pollyea D.A. (2020). Molecular mechanisms mediating relapse following ivosidenib monotherapy in IDH1-mutant relapsed or refractory AML. Blood Adv..

[58] Harding J.J., Lowery M.A., Shih A.H., Schvartzman J.M., Hou S., Famulare C., Patel M., Roshal M., Do R.K., Zehir A. (2018). Isoform Switching as a Mechanism of Acquired Resistance to Mutant Isocitrate Dehydrogenase Inhibition. Cancer Discov..

[59] Amatangelo M.D., Quek L., Shih A., Stein E.M., Roshal M., David M., Marteyn B., Farnoud N.R., DE Botton S., Bernard O. (2017). Enasidenib induces acute myeloid leukemia cell differentiation to promote clinical response. Blood.

[60] Stein E.M., Dinardo C.D., Fathi A.T., Pollyea D.A., Stone R.M., Altman J.K., Roboz G.J., Patel M.R., Collins R., Flinn I.W. (2019). Molecular remission and response patterns in patients with mutant-IDH2 acute myeloid leukemia treated with enasidenib. Blood.

[61] Intlekofer A.M., Shih A.H., Wang B., Nazir A., Rustenburg A.S., Albanese S.K., Patel M., Famulare C., Correa F.M., Takemoto N. (2018). Acquired resistance to IDH inhibition through trans or cis dimer-interface mutations. Nature.

[62] Quek L., David M.D., Kennedy A., Metzner M., Amatangelo M., Shih A., Stoilova B., Quivoron C., Heiblig M., Willekens C. (2018). Clonal heterogeneity of acute myeloid leukemia treated with the IDH2 inhibitor enasidenib. Nat. Med..

[63] Wang F., Morita K., DiNardo C.D., Furudate K., Tanaka T., Yan Y., Patel K.P., MacBeth K.J., Wu B., Liu G. (2021). Leukemia stemness and co-occurring mutations drive resistance to IDH inhibitors in acute myeloid leukemia. Nat. Commun..

[64] Pollyea D.A., Tallman M.S., De Botton S., Kantarjian H.M., Collins R., Stein A.S., Frattini M.G., Xu Q., Tosolini A., See W.L. (2019). Enasidenib, an inhibitor of mutant IDH2 proteins, induces durable remissions in older patients with newly diagnosed acute myeloid leukemia. Leukemia.

[65] Grossmann V., Tiacci E., Holmes A.B., Kohlmann A., Martelli M.P., Kern W., Spanhol-Rosseto A., Klein H.-U., Dugas M., Schindela S. (2011). Whole-exome sequencing identifies somatic mutations of BCOR in acute myeloid leukemia with normal karyotype. Blood.

[66] Damm F., Chesnais V., Nagata Y., Yoshida K., Scourzic L., Okuno Y., Itzykson R., Sanada M., Shiraishi Y., Gelsi-Boyer V. (2013). BCOR and BCORL1 mutations in myelodysplastic syndromes and related disorders. Blood.

[67] Christman J.K. (2002). 5-Azacytidine and 5-aza-2′-deoxycytidine as inhibitors of DNA methylation: Mechanistic studies and their implications for cancer therapy. Oncogene.

[68] Jasielec J., Saloura V., A Godley L. (2014). The mechanistic role of DNA methylation in myeloid leukemogenesis. Leukemia.

[69] Figueroa M.E., Lugthart S., Li Y., Erpelinck-Verschueren C., Deng X., Christos P.J., Schifano E., Booth J., van Putten W., Skrabanek L. (2010). DNA Methylation Signatures Identify Biologically Distinct Subtypes in Acute Myeloid Leukemia. Cancer Cell.

[70] Jones P.A., Baylin S.B. (2002). The fundamental role of epigenetic events in cancer. Nat. Rev. Genet..

[71] Dinardo C.D., Jonas B.A., Pullarkat V., Thirman M.J., Garcia J.S., Wei A.H., Konopleva M., Döhner H., Letai A., Fenaux P. (2020). Azacitidine and Venetoclax in Previously Untreated Acute Myeloid Leukemia. N. Engl. J. Med..

[72] Wei A.H., Döhner H., Pocock C., Montesinos P., Afanasyev B., Dombret H., Ravandi F., Sayar H., Jang J.-H., Porkka K. (2020). Oral Azacitidine Maintenance Therapy for Acute Myeloid Leukemia in First Remission. N. Engl. J. Med..

[73] Emadi A., Faramand R., Carter-Cooper B., Tolu S., Ford L.A., Lapidus R.G., Wetzler M., Wang E.S., Etemadi A., Griffiths E.A. (2015). Presence of isocitrate dehydrogenase mutations may predict clinical response to hypomethylating agents in patients with acute myeloid leukemia. Am. J. Hematol..

[74] Itzykson R., Kosmider O., Cluzeau T., Mansat-De Mas V., Dreyfus F., Beyne-Rauzy O., Quesnel B., Vey N., Gelsi-Boyer V., Raynaud S. (2011). Impact of TET2 mutations on response rate to azacitidine in myelodysplastic syndromes and low blast count acute myeloid leukemias. Leukemia.

[75] Ley T.J., Ding L., Walter M.J., McLellan M.D., Lamprecht T.L., Larson D.E., Kandoth C., Payton J.E., Baty J., Welch J.J. (2010). DNMT3AMutations in Acute Myeloid Leukemia. N. Engl. J. Med..

[76] Thol F., Damm F., Lüdeking A., Winschel C., Wagner K., Morgan M., Yun H., Göhring G., Schlegelberger B., Hoelzer D. (2011). Incidence and Prognostic Influence of DNMT3A Mutations in Acute Myeloid Leukemia. J. Clin. Oncol..

[77] Metzeler K., Walker A., Geyer S., Garzon R., Klisovic R.B., Bloomfield C.D., Blum W., Marcucci G. (2011). DNMT3A mutations and response to the hypomethylating agent decitabine in acute myeloid leukemia. Leukemia.

[78] Welch J.S., Petti A.A., Miller C., Fronick C.C., O’Laughlin M., Fulton R.S., Wilson R.K., Baty J.D., Duncavage E.J., Tandon B. (2016). TP53 and Decitabine in Acute Myeloid Leukemia and Myelodysplastic Syndromes. N. Engl. J. Med..

[79] Döhner H., Dolnik A., Tang L., Seymour J.F., Minden M.D., Stone R.M., Del Castillo T.B., Al-Ali H.K., Santini V., Vyas P. (2018). Cytogenetics and gene mutations influence survival in older patients with acute myeloid leukemia treated with azacitidine or conventional care. Leukemia.

[80] Youle R.J., Strasser A. (2008). The BCL-2 protein family: Opposing activities that mediate cell death. Nat. Rev. Mol. Cell Biol..

[81] Campos L., Rouault J.P., Sabido O., Oriol P., Roubi N., Vasselon C., Archimbaud E., Magaud J.P., Guyotat D. (1993). High expression of bcl-2 protein in acute myeloid leukemia cells is associated with poor response to chemotherapy. Blood.

[82] Bensi L., Longo R., Vecchi A., Messora C., Garagnani L., Bernardi S., Tamassia M.G., Sacchi S. (1995). Bcl-2 oncoprotein expression in acute myeloid leukemia. Haematologica.

[83] Pan R., Hogdal L.J., Benito J.M., Bucci D., Han L., Borthakur G., Cortes J., DeAngelo D.J., DeBose L., Mu H. (2013). Selective BCL-2 Inhibition by ABT-199 Causes On-Target Cell Death in Acute Myeloid Leukemia. Cancer Discov..

[84] Souers A.J., Leverson J.D., Boghaert E.R., Ackler S.L., Catron N.D., Chen J., Dayton B.D., Ding H., Enschede S.H., Fairbrother W.J. (2013). ABT-199, a potent and selective BCL-2 inhibitor, achieves antitumor activity while sparing platelets. Nat. Med..

[85] Konopleva M., Pollyea D.A., Potluri J., Chyla B., Hogdal L., Busman T., McKeegan E., Salem A.H., Zhu M., Ricker J.L. (2016). Efficacy and Biological Correlates of Response in a Phase II Study of Venetoclax Monotherapy in Patients with Acute Myelogenous Leukemia. Cancer Discov..

[86] Wei A.H., Strickland S.A., Hou J.-Z., Fiedler W., Lin T., Walter R.B., Enjeti A., Tiong I.S., Savona M., Lee S. (2019). Venetoclax Combined With Low-Dose Cytarabine for Previously Untreated Patients With Acute Myeloid Leukemia: Results From a Phase Ib/II Study. J. Clin. Oncol..

[87] Wei A.H., Montesinos P., Ivanov V., Dinardo C.D., Novak J., Laribi K., Kim I., Stevens D.A., Fiedler W., Pagoni M. (2020). Venetoclax plus LDAC for newly diagnosed AML ineligible for intensive chemotherapy: A phase 3 randomized placebo-controlled trial. Blood.

[88] Jin S., Cojocari D., Purkal J.J., Popovic R., Talaty N.N., Xiao Y., Solomon L.R., Boghaert E.R., Leverson J.D., Phillips D.C. (2020). 5-Azacitidine Induces NOXA to Prime AML Cells for Venetoclax-Mediated Apoptosis. Clin. Cancer Res..

[89] Chua C.C., Roberts A.W., Reynolds J., Fong C.Y., Ting S.B., Salmon J.M., MacRaild S., Ivey A., Tiong I.S., Fleming S. (2020). Chemotherapy and Venetoclax in Elderly Acute Myeloid Leukemia Trial (CAVEAT): A Phase Ib Dose-Escalation Study of Venetoclax Combined With Modified Intensive Chemotherapy. J. Clin. Oncol..

[90] Chyla B., Daver N., Doyle K., McKeegan E., Huang X., Ruvolo V., Wang Z., Chen K., Souers A., Leverson J. (2018). Genetic biomarkers of sensitivity and resistance to venetoclax monotherapy in patients with relapsed acute myeloid leukemia. Am. J. Hematol..

[91] DiNardo C.D., Tiong I.S., Quaglieri A., MacRaild S., Loghavi S., Brown F.C., Thijssen R., Pomilio G., Ivey A., Salmon J. (2020). Molecular patterns of response and treatment failure after frontline venetoclax combinations in older patients with AML. Blood.

[92] Wang Y.-W., Tsai C., Lin C.-C., Tien F.-M., Chen Y.-W., Lin H.-Y., Yao M., Lin Y.-C., Cheng C.-L., Tang J.-L. (2020). Cytogenetics and mutations could predict outcome in relapsed and refractory acute myeloid leukemia patients receiving BCL-2 inhibitor venetoclax. Ann. Hematol..

[93] Dinardo C.D., Pratz K., Pullarkat V., Jonas B.A., Arellano M., Becker P.S., Frankfurt O., Konopleva M., Wei A.H., Kantarjian H.M. (2018). Venetoclax combined with decitabine or azacitidine in treatment-naive, elderly patients with acute myeloid leukemia. Blood.

[94] Pollyea D.A., DiNardo C.D., Arellano M.L., Pigneux A., Fiedler W., Konopleva M., Rizzieri D.A., Smith B.D., Shinagawa A., Lemoli R.M. (2022). Impact of Venetoclax and Azacitidine in Treatment-Naïve Patients with Acute Myeloid Leukemia and IDH1/2 Mutations. Clin. Cancer Res..

[95] Bisaillon R., Moison C., Thiollier C., Krosl J., Bordeleau M.-E., Lehnertz B., Lavallée V.-P., MacRae T., Mayotte N., Labelle C. (2019). Genetic characterization of ABT-199 sensitivity in human AML. Leukemia.

[96] Chan S.M., Thomas D., Corces-Zimmerman M.R., Xavy S., Rastogi S., Hong W.-J., Zhao F., Medeiros B.C., Tyvoll D.A., Majeti R. (2015). Isocitrate dehydrogenase 1 and 2 mutations induce BCL-2 dependence in acute myeloid leukemia. Nat. Med..

[97] Lachowiez C.A., Loghavi S., Kadia T.M., Daver N., Borthakur G., Pemmaraju N., Naqvi K., Alvarado Y., Yilmaz M., Short N. (2020). Outcomes of older patients with NPM1-mutated AML: Current treatments and the promise of venetoclax-based regimens. Blood Adv..

[98] Lachowiez C.A., Loghavi S., Furudate K., Montalban-Bravo G., Maiti A., Kadia T., Daver N., Borthakur G., Pemmaraju N., Sasaki K. (2021). Impact of splicing mutations in acute myeloid leukemia treated with hypomethylating agents combined with venetoclax. Blood Adv..

[99] Morsia E., McCullough K., Joshi M., Cook J., Alkhateeb H.B., Al-Kali A., Begna K., Elliott M., Hogan W., Litzow M. (2020). Venetoclax and hypomethylating agents in acute myeloid leukemia: Mayo Clinic series on 86 patients. Am. J. Hematol..

[100] Stahl M., Menghrajani K., Derkach A., Chan A., Xiao W., Glass J., King A.C., Daniyan A.F., Famulare C., Cuello B.M. (2021). Clinical and molecular predictors of response and survival following venetoclax therapy in relapsed/refractory AML. Blood Adv..

[101] Pei S., Pollyea D.A., Gustafson A., Stevens B.M., Minhajuddin M., Fu R., Riemondy K.A., Gillen A.E., Sheridan R.M., Kim J. (2020). Monocytic Subclones Confer Resistance to Venetoclax-Based Therapy in Patients with Acute Myeloid Leukemia. Cancer Discov..

[102] Zhang H., Nakauchi Y., Köhnke T., Stafford M., Bottomly D., Thomas R., Wilmot B., McWeeney S.K., Majeti R., Tyner J.W. (2020). Integrated analysis of patient samples identifies biomarkers for venetoclax efficacy and combination strategies in acute myeloid leukemia. Nat. Cancer.

[103] Kontro M., Kumar A., Majumder M.M., Eldfors S., Parsons A., Pemovska T., Saarela J., Yadav B., Malani D., Fløisand Y. (2016). HOX gene expression predicts response to BCL-2 inhibition in acute myeloid leukemia. Leukemia.

[104] Rücker F.G., Schlenk R.F., Bullinger L., Kayser S., Teleanu V., Kett H., Habdank M., Kugler C.-M., Holzmann K., Gaidzik V.I. (2012). TP53 alterations in acute myeloid leukemia with complex karyotype correlate with specific copy number alterations, monosomal karyotype, and dismal outcome. Blood.

[105] Nechiporuk T., Kurtz S.E., Nikolova O., Liu T., Jones C.L., D’Alessandro A., Culp-Hill R., D’Almeida A., Joshi S., Rosenberg M. (2019). The TP53 Apoptotic Network Is a Primary Mediator of Resistance to BCL2 Inhibition in AML Cells. Cancer Discov..

[106] Kim K., Maiti A., Loghavi S., Pourebrahim R., Kadia T.M., Rausch C.R., Furudate K., Daver N.G., Alvarado Y., Ohanian M. (2021). Outcomes of TP53 -mutant acute myeloid leukemia with decitabine and venetoclax. Cancer.

[107] Venugopal S., Shoukier M., Konopleva M., Dinardo C.D., Ravandi F., Short N.J., Andreeff M., Borthakur G., Daver N., Pemmaraju N. (2021). Outcomes in patients with newly diagnosed TP53 -mutated acute myeloid leukemia with or without venetoclax-based therapy. Cancer.

[108] Brinton L.T., Zhang P., Williams K., Canfield D., Orwick S., Sher S., Wasmuth R., Beaver L., Cempre C., Skinner J. (2020). Synergistic effect of BCL2 and FLT3 co-inhibition in acute myeloid leukemia. J. Hematol. Oncol..

[109] Ma J., Zhao S., Qiao X., Knight T., Edwards H., Polin L., Kushner J., Dzinic S.H., White K., Wang G. (2019). Inhibition of Bcl-2 Synergistically Enhances the Antileukemic Activity of Midostaurin and Gilteritinib in Preclinical Models of FLT3-Mutated Acute Myeloid Leukemia. Clin. Cancer Res..

[110] Mali R.S., Zhang Q., DeFilippis R., Cavazos A., Kuruvilla V.M., Raman J., Mody V., Choo E.F., Dail M., Shah N.P. (2020). Venetoclax combines synergistically with FLT3 inhibition to effectively target leukemic cells in FLT3-ITD+ acute myeloid leukemia models. Haematologica.

[111] DiNardo C.D., Maiti A., Rausch C.R., Pemmaraju N., Naqvi K., Daver N.G., Kadia T.M., Borthakur G., Ohanian M., Alvarado Y. (2020). 10-day decitabine with venetoclax for newly diagnosed intensive chemotherapy ineligible, and relapsed or refractory acute myeloid leukaemia: A single-centre, phase 2 trial. Lancet Haematol..

[112] Ramsey H.E., Fischer M.A., Lee T., Gorska A.E., Arrate M.P., Fuller L., Boyd K.L., Strickland S.A., Sensintaffar J., Hogdal L.J. (2018). A Novel MCL1 Inhibitor Combined with Venetoclax Rescues Venetoclax-Resistant Acute Myelogenous Leukemia. Cancer Discov..

[113] Moujalled D.M., Pomilio G., Ghiurau C., Ivey A., Salmon J., Rijal S., MacRaild S., Zhang L., Teh T.-C., Tiong I.-S. (2018). Combining BH3-mimetics to target both BCL-2 and MCL1 has potent activity in pre-clinical models of acute myeloid leukemia. Leukemia.

[114] Hormi M., Birsen R., Belhadj M., Huynh T., Aguilar L.C., Grignano E., Haddaoui L., Guillonneau F., Mayeux P., Hunault M. (2020). Pairing MCL-1 inhibition with venetoclax improves therapeutic efficiency of BH3-mimetics in AML. Eur. J. Haematol..

[115] Wang Q., Wan J., Zhang W., Hao S. (2019). MCL-1 or BCL-xL-dependent resistance to the BCL-2 antagonist (ABT-199) can be overcome by specific inhibitor as single agents and in combination with ABT-199 in acute myeloid leukemia cells. Leuk. Lymphoma.

[116] Bhatt S., Pioso M.S., Olesinski E.A., Yilma B., Ryan J.A., Mashaka T., Leutz B., Adamia S., Zhu H., Kuang Y. (2020). Reduced Mitochondrial Apoptotic Priming Drives Resistance to BH3 Mimetics in Acute Myeloid Leukemia. Cancer Cell.

[117] Han L., Zhang Q., Dail M., Shi C., Cavazos A., Ruvolo V.R., Zhao Y., Kim E., Rahmani M., Mak D.H. (2019). Concomitant targeting of BCL2 with venetoclax and MAPK signaling with cobimetinib in acute myeloid leukemia models. Haematologica.

[118] Kumar B., Garcia M., Weng L., Jung X., Murakami J.L., Hu X., McDonald T., Lin A., Kumar A.R., DiGiusto D.L. (2017). Acute myeloid leukemia transforms the bone marrow niche into a leukemia-permissive microenvironment through exosome secretion. Leukemia.

[119] Wojtuszkiewicz A., Schuurhuis G.J., Kessler F.L., Piersma S.R., Knol J.C., Pham T.V., Jansen G., Musters R.J.P., van Meerloo J., Assaraf Y.G. (2016). Exosomes Secreted by Apoptosis-Resistant Acute Myeloid Leukemia (AML) Blasts Harbor Regulatory Network Proteins Potentially Involved in Antagonism of Apoptosis. Mol. Cell. Proteom..

[120] Mirfakhraie R., Noorazar L., Mohammadian M., Hajifathali A., Gholizadeh M., Salimi M., Sankanian G., Roshandel E., Mehdizadeh M. (2021). Treatment Failure in Acute Myeloid Leukemia: Focus on the Role of Extracellular Vesicles. Leuk. Res..

[121] Stevens B.M., Jones C.L., Pollyea D.A., Culp-Hill R., D’Alessandro A., Winters A., Krug A., Abbott D., Goosman M., Pei S. (2020). Fatty acid metabolism underlies venetoclax resistance in acute myeloid leukemia stem cells. Nat. Cancer.

[122] Abraham A., Matsui W. (2021). Hedgehog Signaling in Myeloid Malignancies. Cancers.

[123] Irvine D.A., Copland M. (2012). Targeting hedgehog in hematologic malignancy. Blood.

[124] Queiroz K.C.S., Ruela-De-Sousa R.R., Fuhler G.M., Aberson H.L., Ferreira C.V., Peppelenbosch M., Spek C.A. (2010). Hedgehog signaling maintains chemoresistance in myeloid leukemic cells. Oncogene.

[125] Cortes J.E., Heidel F.H., Fiedler W., Smith B.D., Robak T., Montesinos P., Candoni A., Leber B., Sekeres M.A., Pollyea D.A. (2020). Survival outcomes and clinical benefit in patients with acute myeloid leukemia treated with glasdegib and low-dose cytarabine according to response to therapy. J. Hematol. Oncol..

[126] Heuser M., Smith B.D., Fiedler W., Sekeres M.A., Montesinos P., Leber B., Merchant A., Papayannidis C., Pérez-Simón J.A., Hoang C.J. (2021). Clinical benefit of glasdegib plus low-dose cytarabine in patients with de novo and secondary acute myeloid leukemia: Long-term analysis of a phase II randomized trial. Ann. Hematol..

[127] Linenberger M.L. (2004). CD33-directed therapy with gemtuzumab ozogamicin in acute myeloid leukemia: Progress in understanding cytotoxicity and potential mechanisms of drug resistance. Leukemia.

[128] Hamann P.R., Hinman L.M., Beyer C.F., Lindh D., Upeslacis J., Flowers D.A., Bernstein I. (2001). An Anti-CD33 Antibody−Calicheamicin Conjugate for Treatment of Acute Myeloid Leukemia. Choice of Linker. Bioconjugate Chem..

[129] Taksin A.-L., Legrand O., Raffoux E., De Revel T., Thomas X., Contentin N., Bouabdallah R., Pautas C., Turlure P., Reman O. (2006). High efficacy and safety profile of fractionated doses of Mylotarg as induction therapy in patients with relapsed acute myeloblastic leukemia: A prospective study of the alfa group. Leukemia.

[130] Lambert J., Pautas C., Terré C., Raffoux E., Turlure P., Caillot D., Legrand O., Thomas X., Gardin C., Gogat-Marchant K. (2018). Gemtuzumab ozogamicin for de novo acute myeloid leukemia: Final efficacy and safety updates from the open-label, phase III ALFA-0701 trial. Haematologica.

[131] Khan N., Hills R.K., Virgo P., Couzens S., Clark N., Gilkes A., Richardson P., Knapper S., Grimwade D., on behalf of the UK NCRI-AML Study Group (2017). Expression of CD33 is a predictive factor for effect of gemtuzumab ozogamicin at different doses in adult acute myeloid leukaemia. Leukemia.

[132] Fournier E., Duployez N., Ducourneau B., Raffoux E., Turlure P., Caillot D., Thomas X., Marceau-Renaut A., Chantepie S.P., Malfuson J.-V. (2020). Mutational profile and benefit of gemtuzumab ozogamicin in acute myeloid leukemia. Blood.

[133] Feldman E.J., Lancet J.E., Kolitz J.E., Ritchie E.K., Roboz G.J., List A., Allen S., Asatiani E., Mayer L.D., Swenson C. (2011). First-In-Man Study of CPX-351: A Liposomal Carrier Containing Cytarabine and Daunorubicin in a Fixed 5:1 Molar Ratio for the Treatment of Relapsed and Refractory Acute Myeloid Leukemia. J. Clin. Oncol..

[134] Feldman E., Kolitz J., Trang J., Liboiron B., Swenson C., Chiarella M., Mayer L., Louie A., Lancet J. (2012). Pharmacokinetics of CPX-351; a nano-scale liposomal fixed molar ratio formulation of cytarabine:daunorubicin, in patients with advanced leukemia. Leuk. Res..

[135] (2017). Administration, U.S.F.a.D. Drug Approval Package: VYXEOS (daunorubicin and cytarabine). FDA webpage. https://www.accessdata.fda.gov/drugsatfda_docs/nda/2017/209401Orig1s000TOC.cfm.

[136] Agency E.M. Vyxeos liposomal (previously known as Vyxeos) EMA 2018. https://www.ema.europa.eu/en/medicines/hu-1102man/EPAR/vyxeos-liposomal.

[137] Lancet J.E., Uy G.L., Cortes J.E., Newell L.F., Lin T.L., Ritchie E.K., Stuart R.K., Strickland S.A., Hogge D., Solomon S.R. (2018). CPX-351 (cytarabine and daunorubicin) Liposome for Injection Versus Conventional Cytarabine Plus Daunorubicin in Older Patients With Newly Diagnosed Secondary Acute Myeloid Leukemia. J. Clin. Oncol..

[138] Chiche E., Rahmé R., Bertoli S., Dumas P.-Y., Micol J.-B., Hicheri Y., Pasquier F., Peterlin P., Chevallier P., Thomas X. (2021). Real-life experience with CPX-351 and impact on the outcome of high-risk AML patients: A multicentric French cohort. Blood Adv..

[139] Rautenberg C., Stölzel F., Röllig C., Stelljes M., Gaidzik V., Lauseker M., Kriege O., Verbeek M., Unglaub J.M., Thol F. (2021). Real-world experience of CPX-351 as first-line treatment for patients with acute myeloid leukemia. Blood Cancer J..

[140] Guolo F., Fianchi L., Minetto P., Clavio M., Gottardi M., Galimberti S., Rizzuto G., Rondoni M., Bertani G., Dargenio M. (2020). CPX-351 treatment in secondary acute myeloblastic leukemia is effective and improves the feasibility of allogeneic stem cell transplantation: Results of the Italian compassionate use program. Blood Cancer J..

[141] Cluzeau T., Loschi M., Fenaux P., Komrokji R., Sallman D.A. (2021). Personalized Medicine for TP53 Mutated Myelodysplastic Syndromes and Acute Myeloid Leukemia. Int. J. Mol. Sci..

[142] Lambert J.M., Gorzov P., Veprintsev D., Söderqvist M., Segerbäck D., Bergman J., Fersht A.R., Hainaut P., Wiman K.G., Bykov V.N. (2009). PRIMA-1 Reactivates Mutant p53 by Covalent Binding to the Core Domain. Cancer Cell.

[143] Degtjarik O., Golovenko D., Diskin-Posner Y., Abrahmsén L., Rozenberg H., Shakked Z. (2021). Structural basis of reactivation of oncogenic p53 mutants by a small molecule: Methylene quinuclidinone (MQ). Nat. Commun..

[144] Cluzeau T., Sebert M., Rahmé R., Cuzzubbo S., Lehmann-Che J., Madelaine I., Peterlin P., Bève B., Attalah H., Chermat F. (2021). Eprenetapopt Plus Azacitidine in TP53-Mutated Myelodysplastic Syndromes and Acute Myeloid Leukemia: A Phase II Study by the Groupe Francophone des Myélodysplasies (GFM). J. Clin. Oncol..

[145] Sallman D.A., DeZern A.E., Garcia-Manero G., Steensma D.P., Roboz G.J., Sekeres M.A., Cluzeau T., Sweet K.L., McLemore A., McGraw K.L. (2021). Eprenetapopt (APR-246) and Azacitidine in TP53-Mutant Myelodysplastic Syndromes. J. Clin. Oncol..

[146] Khoury J.D., Tashakori M., Yang H., Loghavi S., Wang Y., Wang J., Piya S., Borthakur G. (2020). Pan-RAF Inhibition Shows Anti-Leukemic Activity in *RAS*-Mutant Acute Myeloid Leukemia Cells and Potentiates the Effect of Sorafenib in Cells with *FLT3* Mutation. Cancers.

[147] Tambe M., Karjalainen E., Vähä-Koskela M., Bulanova D., Gjertsen B.T., Kontro M., Porkka K., Heckman C.A., Wennerberg K. (2020). Pan-RAF inhibition induces apoptosis in acute myeloid leukemia cells and synergizes with BCL2 inhibition. Leukemia.

[148] Morales M.L., Arenas A., Ortiz-Ruiz A., Leivas A., Rapado I., Rodríguez-García A., Castro N., Zagorac I., Quintela-Fandino M., López G.G. (2019). MEK inhibition enhances the response to tyrosine kinase inhibitors in acute myeloid leukemia. Sci. Rep..

[149] Lu H., Li Z.-Y., Ding M., Liang C., Weng X.-Q., Sheng Y., Wu J., Cai X. (2021). Trametinib enhances ATRA-induced differentiation in AML cells. Leuk. Lymphoma.

[150] Jain N., Curran E., Iyengar N.M., Diaz-Flores E., Kunnavakkam R., Popplewell L., Kirschbaum M.H., Karrison T., Erba H.P., Green M. (2013). Phase II Study of the Oral MEK Inhibitor Selumetinib in Advanced Acute Myelogenous Leukemia: A University of Chicago Phase II Consortium Trial. Clin. Cancer Res..

[151] Ragon B.K., Odenike O., Baer M.R., Stock W., Borthakur G., Patel K., Han L., Chen H., Ma H., Joseph L. (2019). Oral MEK 1/2 Inhibitor Trametinib in Combination With AKT Inhibitor GSK2141795 in Patients With Acute Myeloid Leukemia With RAS Mutations: A Phase II Study. Clin. Lymphoma Myeloma Leuk..

[152] Issa G.C., Ravandi F., DiNardo C.D., Jabbour E., Kantarjian H.M., Andreeff M. (2021). Therapeutic implications of menin inhibition in acute leukemias. Leukemia.

[153] Borate U., Esteve J., Porkka K., Knapper S., Vey N., Scholl S., Garcia-Manero G., Wermke M., Janssen J., Traer E. (2019). Phase Ib Study of the Anti-TIM-3 Antibody MBG453 in Combination with Decitabine in Patients with High-Risk Myelodysplastic Syndrome (MDS) and Acute Myeloid Leukemia (AML). Blood.

[154] Mimitou E.P., Lareau C.A., Chen K.Y., Zorzetto-Fernandes A.L., Hao Y., Takeshima Y., Luo W., Huang T.-S., Yeung B.Z., Papalexi E. (2021). Scalable, multimodal profiling of chromatin accessibility, gene expression and protein levels in single cells. Nat. Biotechnol..

[155] Lareau C.A., Ludwig L.S., Muus C., Gohil S.H., Zhao T., Chiang Z., Pelka K., Verboon J.M., Luo W., Christian E. (2020). Massively parallel single-cell mitochondrial DNA genotyping and chromatin profiling. Nat. Biotechnol..

[156] Velten L., Story B.A., Hernández-Malmierca P., Raffel S., Leonce D.R., Milbank J., Paulsen M., Demir A., Szu-Tu C., Frömel R. (2021). Identification of leukemic and pre-leukemic stem cells by clonal tracking from single-cell transcriptomics. Nat. Commun..

[157] Stetson L.C., Balasubramanian D., Ribeiro S.P., Stefan T., Gupta K., Xu X., Fourati S., Roe A., Jackson Z., Schauner R. (2021). Single cell RNA sequencing of AML initiating cells reveals RNA-based evolution during disease progression. Leukemia.

[158] Miles L.A., Bowman R.L., Merlinsky T.R., Csete I.S., Ooi A.T., Durruthy-Durruthy R., Bowman M., Famulare C., Patel M.A., Mendez P. (2020). Single-cell mutation analysis of clonal evolution in myeloid malignancies. Nature.

[159] Morita K., Wang F., Jahn K., Hu T., Tanaka T., Sasaki Y., Kuipers J., Loghavi S., Wang S.A., Yan Y. (2020). Clonal evolution of acute myeloid leukemia revealed by high-throughput single-cell genomics. Nat. Commun..

